# Erstfraktur bei Patienten mit Osteoporose: Womit beginnt die „Frakturkarriere“ unserer Osteoporosepatienten?

**DOI:** 10.1007/s00113-025-01535-4

**Published:** 2025-01-31

**Authors:** Steffi S. I. Falk

**Affiliations:** Klinik für Unfall‑, Hand- und Wiederherstellungschirurgie, Universitätsmedizin Rostock, Schillingallee 35, 18057 Rostock, Deutschland

**Keywords:** Sarkopenie, Geriatrische Patienten, Knochenschwund, Folgefraktur, Niedrigenergetische Traumata, Sarcopenia, Geriatric patients, Bone loss, Secondary fracture, Low-energy trauma

## Abstract

**Hintergrund:**

Unter Berücksichtigung der Tatsachen, dass die erste osteoporotische Fraktur weitere nach sich zieht und die genauen Ursachen der typischen Frakturkaskade bisher noch ungeklärt sind, werfen sich die Fragen auf, an welchem Punkt diese Kaskade ihren Anfang nimmt und mit welcher Fraktur.

**Methodik:**

Über den Untersuchungszeitraum von 12 Monaten wurden alle Patienten mit einer Fraktur der langen Röhrenknochen konsekutiv gescreent. Alle Teilnehmer wurden hinsichtlich vorliegender Risikofaktoren (inkl. Vorfrakturen) gemäß gültiger DVO-Leitlinie befragt. Anhand der vorliegenden Risikofaktoren erfolgte die Berechnung des Frakturrisikos, auf dessen Basis die Diagnose und auch die Therapieindikation erfolgte.

**Ergebnisse:**

Insgesamt konnten 613 Patienten in die Studie eingeschlossen werden. Hierunter bestand bei 378 Patienten die Indikation zur medikamentösen Osteoporosetherapie. In dieser Gruppe wurden 182 Vorfrakturen berichtet. Unter diesen Vorfrakturen war die distale Radiusfraktur mit 54 Patienten am häufigsten vertreten, gefolgt von den hüftgelenknahen Frakturen (*n* = 40) und proximalen Humerusfrakturen (*n* = 27). Bei einer Aufteilung der Daten nach Geschlecht zeigt sich erwartungsgemäß ein verändertes Bild. In Übereinstimmung mit den Inzidenzen lässt sich feststellen, dass Männer weniger distale Radiusfrakturen und dafür mehr hüftgelenknahe Frakturen aufweisen.

**Diskussion:**

Die Ergebnisse legen nahe, dass für Frauen mit distaler Radiusfraktur eine gute Möglichkeit besteht, frühzeitig eine Diagnose und nachfolgend auch eine Therapie bezüglich der Osteoporose einzuleiten. Bei Männern hingegen manifestierten sich hüftgelenknahe Frakturen am häufigsten als Erstfrakturen, sodass durch Vorfrakturen kein frühzeitiger Hinweis auf das Vorliegen einer Osteoporose abgeleitet werden konnte.

## Abstract (en)

**Background:**

Given that a primary osteoporotic fracture can result in subsequent fractures and that the precise etiology of the typical fracture cascade remains uncertain, it is pertinent to determine the initiating factor and the specific fracture involved in this cascade.

**Method:**

All patients with a fracture of a long bone were consecutively screened over the 12-month study period. All participants were asked about existing risk factors (including previous fractures) in accordance with the applicable guidelines of the Umbrella Organization Osteology (DVO). The existing risk factors were employed to calculate the fracture risk and this formed the basis of the diagnosis and indications for treatment.

**Results:**

A total of 613 patients were included in the study. Of the total number of patients 378 were deemed to require pharmacological treatment for osteoporosis. In this cohort 182 fractures were reported. Among the previous fractures distal radius fractures were the most common, occurring in 54 patients followed by fractures of the proximal femur (*n* = 40) and proximal humerus (*n* = 27). As anticipated, a breakdown of the data by gender revealed a different picture. In line with the incidences it can be seen that men have fewer distal radius fractures and more fractures close to the hip joint.

**Discussion:**

The results indicate that women with a distal radius fracture have the potential to facilitate an early diagnosis and subsequent treatment for osteoporosis. In contrast, in men fractures in the region of the hip joint were the most common initial presentation, precluding the possibility of deriving an early indication of the presence of osteoporosis from previous fractures.

Unter Berücksichtigung der Tatsachen, dass die erste osteoporotische Fraktur weitere nach sich zieht und die genauen Ursachen der typischen Frakturkaskade bisher noch ungeklärt sind, muss eruiert werden, an welchem Punkt diese Kaskade ihren Anfang nimmt und mit welcher Fraktur. Die hier vorgestellte Studie hat über einen Zeitraum von 12 Monaten unter 613 unfallchirurgischen Patienten untersucht, an welchem Punkt die typische Frakturkaskade ihren Anfang nimmt und mit welcher Fraktur sie beginnt.

## Einleitung

Die Osteoporose ist eine Erkrankung, die durch eine verminderte Knochenmasse und eine verringerte Knochenqualität gekennzeichnet ist. Beide Faktoren resultieren in einer erhöhten Fragilität des Knochens, was das Risiko für Frakturen signifikant erhöht [1]. Folgen dieser Knochenfrakturen sind eine erhebliche Einschränkung der Lebensqualität für den betroffenen Patienten sowie eine volkswirtschaftliche Belastung. Die International Osteoporosis Foundation (IOF) bezifferte die Anzahl der Osteoporose-Erkrankten in Deutschland zuletzt mit 5,3 Mio. [2]. Gemäß dem Hamburg Center for Health Economics stellt Osteoporose mit den zugehörigen Frakturen die häufigste Erkrankung bei Frauen über 50 Jahren dar. In dieser Gruppe ist Osteoporose somit häufiger als Herz-Kreislauf-Erkrankungen [3].

Osteoporose manifestiert sich bei den Betroffenen häufig erst durch sog. Indikatorfrakturen. In der Leitlinie des Dachverband Osteologie (DVO) von 2023 werden hier die Wirbelkörperfraktur und die proximale Femurfraktur genannt [4]. Unter Berücksichtigung der Tatsache, dass die Wahrscheinlichkeit, im weiteren Leben einen Knochenbruch zu erleiden, für Frauen jenseits des 50. Lebensjahres mit bis zu 46 % und auch für Männer ein Frakturrisiko über alle Frakturentitäten bis zu 22 % angegeben wird, kann die Gruppe der Betroffenen als groß bezeichnet werden [5]. Allein die Anzahl der osteoporosebedingten Frakturen wird für Deutschland jährlich auf 725.000 geschätzt. Eine Differenzierung der häufigsten Frakturen ist in Tab. 1 dargestellt.

**Tab. 1:** Aufschlüsselung des Lebenszeitrisikos für die häufigsten Frakturen, getrennt für Männer und Frauen ab dem 50. Lebensjahr. (Modifiziert nach Johnell und Kanis [5])

	Frauen (in %)	Männer (in %)
Distale Radiusfrakturen	13–21	3–5
Hüftgelenknahe Frakturen	11–23	3–11
Wirbelkörperfrakturen	3–16	1–8
Gesamtrisiko über alle Frakturen	40–46	13–22

Zu den klassischen osteoporoseassoziierten Knochenbrüchen zählen die distale Radiusfraktur, die proximale Humerusfraktur sowie hüftgelenknahe Frakturen und Wirbelkörperfrakturen. Bezüglich dieser Frakturen ist bekannt, dass ihre Inzidenz mit dem Alter ansteigt [6]. Die Literatur bietet zwar Inzidenzen zu den einzelnen Knochenbrüchen, jedoch nur vereinzelte Studien zur Folgefraktur. Die typische Kaskade der Frakturen ist bislang noch nicht vollständig aufgeklärt, insbesondere die Frage, an welcher Stelle sie ihren Anfang nimmt, ist noch ungeklärt.

Aus Voruntersuchungen von Balasubramanian et al. [7], Söreskog et al. [8] sowie Chen et al. [9] sowie aus der Leitlinie zur Osteoporose [4] ist bekannt, dass die erste Fraktur das Risiko für weitere Frakturen erhöht und somit den Grundstein für eine „Frakturkarriere“ legt. Das Risiko für Folgefrakturen, unabhängig von der Frakturentität, erhöht sich gemäß aktuellen Angaben in der Literatur für einen Zeitraum von 10 Jahren und bleibt für die „major osteoporotic fractures“ (MOF; distale Radiusfraktur, proximale Humerusfraktur, Wirbelkörperfrakturen und hüftgelenknahe Frakturen) ein Leben lang bestehen. Das höchste Risiko für eine Folgefraktur besteht in den ersten 12 Monaten. Innerhalb dieses Zeitraums erleiden 10 % unserer Patienten einen erneuten Knochenbruch. Nach einem Zeitraum von 2 Jahren liegt die Rate bei 18 % und nach 5 Jahren bei etwa jedem dritten Patienten [7]. Bislang beschränken sich die Studien zumeist auf die Untersuchung von Einflüssen zwischen 2 Frakturentitäten; über Frakturkaskaden ist wenig bekannt. Chen et al. [9] konnten nachweisen, dass insbesondere distale Radiusfrakturen das Risiko für eine hüftgelenknahe Fraktur erhöhen, wobei dieser Effekt insbesondere in den ersten 4 Wochen nach der Femurfraktur zu beobachten ist. Demgegenüber ist das Risiko für Wirbelkörperfrakturen insbesondere bei Vorliegen von Wirbelkörperfrakturen erhöht. Dieses Risiko nimmt mit zunehmendem Schweregrad der Wirbelkörperfraktur zu.

Des Weiteren konnten Balasubramanian et al. [7] demonstrieren, dass Wirbelkörperfrakturen das höchste Risiko für Folgefrakturen unter allen Frakturentitäten aufweisen. Das Risiko einer weiteren Fraktur innerhalb der nächsten 5 Jahre wurde mit etwa 35 % angegeben. Für die 9 untersuchten Frakturgruppen konnte nach 5 Jahren bei Patienten über 75 Jahren durchweg eine Wahrscheinlichkeit für eine Folgefraktur über 20 % festgestellt werden. Des Weiteren ist zu berücksichtigen, dass nicht ausgeschlossen werden kann, dass die untersuchten Patienten bereits vor der Untersuchung andere Frakturen erlitten hatten. Diese Daten wurden nicht erhoben. Die Autoren sind folglich in der Lage, eine Aussage zur ersten Folgefraktur, jedoch nicht zur Startfraktur in dieser Frakturkette zu treffen.

Vor dem Hintergrund, dass in Deutschland weiterhin eine Therapielücke für die Osteoporose von 80 % besteht, zielte diese Untersuchung darauf ab, die Frage zu beantworten, mit welcher Fraktur die „Frakturkarriere“, die die Lebensqualität unserer Patienten stark mindert, beginnt. In anderen Worten: Welcher Zeitpunkt ist besonders geeignet, um eine Osteoporosediagnose und -therapie zu beginnen, um möglichst viele Folgefrakturen zu verhindern?

## Methodik

Im Rahmen einer prospektiven Studie wurden alle Patienten mit operativ zu versorgenden Frakturen der großen Röhrenknochen über einen Zeitraum von 12 Monaten auf eine Teilnahme gescreent. Die Einschlusskriterien umfassten ein Mindestalter von 45 Jahren sowie eine Fraktur, die auf Basis eines niedrigenergetischen Traumas, wie beispielsweise einem Sturz aus dem Stand, resultierte. Die Einschlusskriterien berücksichtigen die Gruppe der niedrigenergetischen Wirbelkörperfrakturen nicht. Dies erfolgt, da Frakturen der langen Röhrenknochen direkt zu Diagnostik und Therapie führen, während osteoporoseassoziierte Wirbelkörperfrakturen in nichtunerheblichem Maße gar nicht oder erst später erkannt werden [10–12]. Um diese Unschärfe zu vermeiden, fokussierte diese Studie die Frakturen der langen Röhrenknochen. Des Weiteren wurden Patienten, die eine pathologische Fraktur aufgrund einer Tumorerkrankung oder eine dialysepflichtige Niereninsuffizienz aufwiesen, von der Studie ausgeschlossen. Die Teilnahme an der Studie erfolgte nach schriftlicher Einwilligung der Patienten. Im Anschluss erfolgte die Erhebung der Frakturanamnese sowie möglicher Risikofaktoren für eine Osteoporose. Die Risikofaktoren wurden entsprechend der zum Studienzeitpunkt gültigen Fassung der Leitlinie zur Osteoporose der DVO festgelegt [13]. Des Weiteren wurde für die Studienteilnehmer die bestehende Frakturwahrscheinlichkeit mittels FRAX® berechnet [14]. Um einen Bias durch eine nichtbestehende Osteoporose zu vermeiden, erfolgt die Auswertung der Frakturen im Rahmen der Frakturkarriere nur für Patienten mit bestehender Indikation für eine Osteoporosetherapie. Die Therapieschwelle wurde für das Risiko proximaler Femurfrakturen mit 3 % angenommen, während für MOF ein Schwellenwert von 20 % galt [15]. Da das Risikomerkmal der niedrigenergetischen Fraktur bei allen Patienten vorlag und einen großen Einfluss auf den Risikowert hat, erfolgte die Berechnung des FRAX®-Wertes ohne die aktuelle Fraktur. Dies sollte die falsch-positive Identifikation von Patienten mit Osteoporose senken. Da für die betrachteten Patienten keine DXA-Messungen vorliegen, erfolgte somit die Diagnose einer Osteoporose in dieser Studie für alle Patienten mit einem FRAX®-Wert von 3 % für proximale Femurfrakturen oder 20 % für MOF.

Ebenso wurde bei dem hohen Anteil der Frauen im Patientenkollektiv eine geschlechtergetrennte Auswertung der Vorfrakturen vorgenommen. Die statistische Berechnung zum möglichen Altersunterschied der Geschlechter erfolgte mittels SPSS Version 28 mittels Mann-Whitney-U-Test bei nicht normal verteilten Daten.

## Ergebnisse

**Abb. 1:**
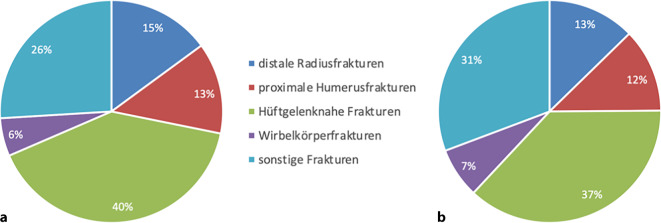
Verteilung der aktuellen Frakturentitäten in der untersuchten Patientengruppe. **a** Subgruppe mit Vorfrakturen, **b** Gruppe ohne Vorfrakturen

Im Zeitraum April 2021 bis April 2022 konnten insgesamt 613 Patienten mit niedrigenergetischen Frakturen in diese Studie eingeschlossen werden. Abb. 1 gibt einen Überblick über die aktuelle Fraktur, die zur stationären Therapie geführt hat. In Übereinstimmung mit den erhobenen Risikofaktoren erfüllten 479 Patienten die Voraussetzungen für die Durchführung einer Basisdiagnostik für Osteoporose gemäß den Leitlinien der DVO. Der anhand des FRAX®-Wertes und der hier festgelegten Definition für Osteoporose lag bei 378 Patienten dieser Gruppe eine Osteoporose vor. Unter diesen 378 Patienten berichteten 177 über eine Vorfraktur (47 %). Das Durchschnittsalter der Patientengruppen mit Therapieindikation lag bei 81 Jahren (Tab. 2).

**Tab. 2:** Demografische Details der betrachteten Patienten mit einer Therapieindikation bei manifester Osteoporose

	Mit Vorfraktur	Ohne Vorfraktur	Gesamt
Anzahl	177 (47 %)	201 (53 %)	378
Alter (in Jahren)	82 (48–97)	80 (59–97)	81 (48–97)
Anteil Frauen	155 (88 %)	158 (79 %)	313 (83 %)

Innerhalb der Gruppe der 177 Patienten mit Vorfrakturen wurden insgesamt 182 Frakturen berichtet. Von den männlichen Teilnehmern (*n* = 22) wurden insgesamt 22 Frakturen berichtet, während es bei den Frauen 160 waren. Die distale Radiusfraktur stellte die häufigste Fraktur in dieser Patientengruppe dar (*n* = 54; 29 %). Dabei handelte es sich um eine klassische osteoporoseassoziierte Fraktur. Die proximale Femurfraktur (*n* = 40; 22 %) stand an zweiter Stelle, gefolgt von der proximalen Humerusfraktur (*n* = 27; 15 %).

**Abb. 2:**
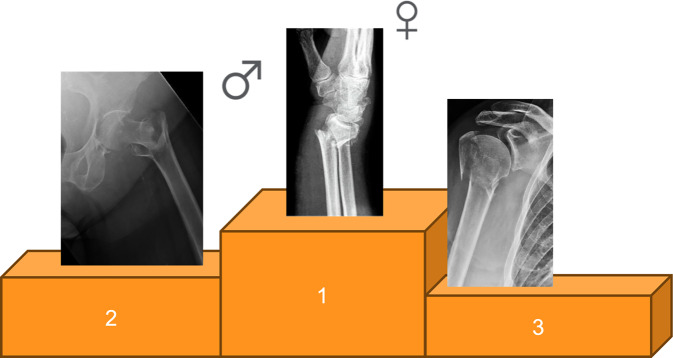
Die häufigsten Erstfrakturen der untersuchten Patienten, mit Markierung der häufigsten Fraktur bezogen auf das Geschlecht

Bei einer getrennten Betrachtung der Geschlechter zeigt sich, dass die distale Radiusfraktur bei Frauen mit *n* = 52 die höchste Prävalenz aufweist. Bei Männern hingegen stellt die proximale Femurfraktur mit *n* = 8 die häufigste Diagnose dar (Abb. 2 und 3). Es konnte kein signifikanter Unterschied im Alter zwischen den Geschlechtern der betrachteten Patienten festgestellt werden (*p* = 0,592). Das Durchschnittsalter der befragten Frauen betrug 82 Jahre, das der Männer 80 Jahre.

**Abb. 3:**
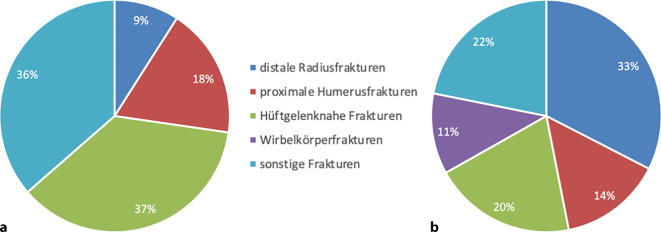
Verteilung der ersten Vorfraktur zwischen den Geschlechtern: **a** Männer, **b** Frauen

## Diskussion

Die erhobenen Ergebnisse mit der Häufigkeit der „major osteoporotic fractures“ – distale Radiusfraktur und proximale Femurfraktur – als häufigste Einstiegsfraktur in die Frakturserie der Osteoporose bestätigen die besondere Relevanz dieser Frakturentitäten im Kontext der Osteoporose.

Die betrachtete Patientengruppe entspricht mit einem Alter von durchschnittlich 81 Jahren dem erwarteten für Patienten mit Osteoporose und zugehöriger Fraktur. Gemäß der Leitlinie der DVO zeigt sich eine Verdoppelung der Inzidenz von „major osteoporotic fractures“ mit jeder weiteren Lebensdekade ab dem 50. Lebensjahr. Diese Steigerung der Inzidenz persistiert bis zu einem Alter von 90 Jahren. Daher lässt sich für die betrachtete Patientengruppe vermuten, dass ein Großteil der Patienten bereits mindestens eine Fraktur erlitten hat. Dies macht eine Erhebung früherer Frakturen zu einer sinnvollen und indizierten Maßnahme. Diese Annahme wird durch die Resultate bestätigt, wonach 47 % der hier eingeschlossenen Patienten eine positive Anamnese für Vorfrakturen aufweisen.

Zudem spiegelt der größere Anteil der Frauen auch das beschriebene größere Patientenkollektiv von Osteoporosebetroffenen wider. Aus der Literatur ist bekannt, dass Frauen früher an Osteoporose erkranken [13]. Zudem stellen sie in der alternden Bevölkerung den größeren Anteil dar und werden aktuell älter als Männer [16, 17]. Insofern war ein größerer Anteil des weiblichen Geschlechts zu erwarten. Auch die Verteilung der zu behandelnden Frakturen spiegelt die Rangfolge der bestehenden Frakturinzidenzen wider. In diesem Kontext sei darauf verwiesen, dass hüftgelenknahe Frakturen sowohl pertrochantäre als auch mediale Schenkelhalsfrakturen umfassen und somit in dieser Auswertung häufiger auftreten als die distale Radiusfraktur, die sonst am häufigsten auftritt. Ebenso muss berücksichtigt werden, dass für die distale Radiusfraktur eine konservative Therapieoption besteht; diese ist für pertrochantäre Femurfrakturen so nicht gegeben und besteht für mediale Schenkelhalsfrakturen nur sehr eingeschränkt. Unter Berücksichtigung der genannten Voraussetzungen kann die vorliegende Patientengruppe als repräsentativ und aussagekräftig bezeichnet werden.

Die distale Radiusfraktur stellt die häufigste Fraktur des Menschen dar und ist auch die häufigste Fraktur in der untersuchten Klientel. Zudem hat die distale Radiusfraktur jenseits des Kindesalters ihren Altersgipfel zwischen 65 und 85 Jahren, was dem Durchschnittsalter entspricht. Somit war sie als häufigste Diagnose bei den zu behandelnden Frakturen zu erwarten. Neu ist der Beleg, dass entsprechend dem Altersgipfel die Radiusfraktur nicht nur zu den häufigsten Frakturen zählt, sondern ihre Funktion als „Wächterfraktur“ oder „Startfraktur“ der Frakturkarriere einnimmt. Unter Berücksichtigung der Inzidenzen, des Altersgipfels sowie der Tatsache, dass Frakturen das Risiko für Folgefrakturen erhöhen, war dies nicht überraschend. Allerdings konnte dies nach den Recherchen der Autoren noch nicht in der Literatur belegt werden.

Die geschlechterspezifische Betrachtung der Ergebnisse erweist sich als besonders interessant. Aufgrund des hohen Anteils der Frauen in dieser Gruppe bleibt in dieser Gruppe die Reihenfolge unverändert. Für Männer hingegen zeigt sich ein anderes Bild: Hier ist die hüftgelenknahe Fraktur an erster Stelle zu verorten. Diese Auffälligkeit stellt eine Neuigkeit dar, über die bislang noch keine Berichte existieren.

Das Ergebnis muss hinsichtlich der Wirbelkörperfrakturen jedoch mit Vorsicht interpretiert werden, da auch im untersuchten Kollektiv von einer Dunkelziffer im Sinne nichterkannter Wirbelkörperfrakturen, die dieses Ergebnis beeinflussen könnten, ausgegangen werden muss. In ihrer Untersuchung ermittelten El Maghraoui et al. eine Inzidenz für stille Wirbelkörperfrakturen von 40 % im Kollektiv der asymptomatischen postmenopausalen Frauen (*n* = 908) [10]. Unter Berücksichtigung der Untersuchungen von Balsubramanian et al. [7] stellt die nichterkannte stille Wirbelkörperfraktur die Hauptlimitation dar, da Balsubramanian et al. zeigen konnten, dass die Wirbelkörperfrakturen unter allen Frakturen das höchste Risiko für eine Folgefraktur hatten. Die Inzidenz für Wirbelkörperfrakturen liegt in der Gruppe der 50- bis 60-jährigen Frauen bei 121/100.000. Die Inzidenz für distale Radiusfrakturen ist in dieser Altersgruppe mit 115/100.000 etwas niedriger. Somit lässt sich ableiten, dass die Wahrscheinlichkeit für das Auftreten einer distalen Radiusfraktur höher ist als die einer Wirbelkörperfraktur, was die Limitation der nichterhobenen stillen Wirbelkörperfraktur abschwächt.

Der berichtete Anteil der Patienten mit Vorfrakturen liegt höher als in der simulierten Berechnung von Hernlund et al. [11]. Hernlund et al. kamen in ihrer simulierten Hochrechnung in der Gruppe der Patienten mit Wirbelkörperfrakturen und hüftgelenknahen Frakturen auf 30 % Vorfrakturen.

Die Laufzeit von 12 Monaten ermöglicht, eine Verzerrung durch jahreszeitliche Effekte auszuschließen, was die Aussagekraft der Studie deutlich erhöht. Des Weiteren ist die Studie mit einer Teilnehmerzahl über 300 ausreichend groß, um Rückschlüsse auf das Gesamtkollektiv der Patienten mit Folgefrakturen zuzulassen.

### Limitierung

Die von der WHO genutzte Definition der messtechnischen Osteoporose basiert auf DXA-Messwerten. In den Leitlinien hat sich jedoch ein Ansatz durchgesetzt, der das Risiko für eine osteoporotische Fraktur in den Mittelpunkt stellt. Das beschriebene Vorgehen erweist sich insbesondere bei fehlendem Zugang zu einer DXA-Messung unter Nutzung eines Risiko-Scores wie FRAX® oder des DVO-Risiko-Scores [4, 18] als geeignet. Zusätzlich wird dadurch eine gute Vergleichbarkeit mit vorhandenen Daten erzielt. Zudem belegen Studien die Korrelation von FRAX® und DXA-Messungen, auch wenn die FRAX®-Risikowertberechnung ohne Eingabe von DXA-Messwerten erfolgt [19]. Die stärkere Gewichtung von Risikofaktoren bei der Entscheidung über die Diagnose belegt auch die neue DVO-Leitlinie [4], welche für hohe Risikokonstellationen keine DXA-Messung für die Therapieentscheidung vorsieht.

Die Studie basiert auf einer Stichprobe von Patienten mit operativ zu versorgenden Frakturen und ist daher nicht repräsentativ für Rückschlüsse auf die Gesamtbevölkerung. Unter Berücksichtigung der Tatsache, dass eine bestehende Fraktur das Risiko für Folgefrakturen erhöht, stellt die betrachtete Patientengruppe genau die benötigte Gruppe dar – Patienten mit interventionspflichtiger Folgefraktur. Die Betrachtung eben jener Gruppe der Patienten mit erneuter Fraktur erlaubt die Schlussfolgerung, dass die Ergebnisse zumindest einen klaren Hinweis auf die Erstfraktur für Patienten mit interventionspflichtigen Folgefrakturen aufzeigen. Insofern wurde genau die Patientengruppe, die am meisten von einer Intervention nach einer ersten Fraktur profitieren würde, erfasst.

### Schlussfolgerung

Die Analyse zeigt, dass sich bei Frauen die klassische MOF als Startfraktur mit dem größten Anteil distaler Radiusfrakturen manifestiert, während bei Männern ebenfalls MOF dominieren, allerdings die hüftgelenknahe Fraktur am häufigsten auftritt. Dies ist insbesondere in Anbetracht der Mortalität der hüftgelenknahen Frakturen eine überraschende Erkenntnis. Die Ergebnisse untermauern aus Sicht der Autoren die geschlechterspezifische Betrachtungsweise der Osteoporose sowie die Schlüsselposition der distalen Radiusfraktur bei Frauen und insbesondere der hüftgelenknahen Fraktur bei Männern.

## Fazit für die Praxis

- Diagnostik und Therapie der Osteoporose erfolgen weiterhin zu selten.
- Für Frauen mit distalen Radiusfrakturen sollte eine Osteoporosediagnostik erfolgen.
- Für Patienten mit hüftgelenknahen Frakturen sollte eine zeitnahe Osteoporosetherapie erfolgen.

## Data Availability

Die erhobenen Datensätze können auf begründete Anfrage in anonymisierter Form beim korrespondierenden Autor angefordert werden. Die Daten befinden sich auf einem Datenspeicher am Universitätsklinikum Rostock in der Klinik für Unfall‑, Hand- und Wiederherstellungschirurgie.

## References

[1] Svedbom A, Hernlund E, Ivergård M et al (2013) Osteoporosis in the European Union: a compendium of country-specific reports. Arch Osteoporos. 10.1007/s11657-013-0137-0 24113838 10.1007/s11657-013-0137-0 PMC3880492

[2] Kanis JA, Norton N, Harvey NC et al (2021) SCOPE 2021: a new scorecard for osteoporosis in Europe. Arch Osteoporos. 10.1007/s11657-020-00871-9 34080059 10.1007/s11657-020-00871-9 PMC8172408

[3] Ärzteblatt Knochenbrüche durch Osteoporose verursachen hohe Kosten. https://www.aerzteblatt.de/nachrichten/62470/Knochenbrueche-durch-Osteoporose-verursachen-hohe-Kosten. Zugegriffen: 27. März 2024

[4] Osteologie DD Prophylaxe, Diagnostik und Therapie der Osteoporose bei postmenopausalen Frauen und bei Männern ab dem 50.Lebensjahr. https://register.awmf.org/assets/guidelines/183-001l_S3_Prophylaxe-Diagnostik-Therapie-der-Osteoporose_2023-11.pdf. Zugegriffen: 27. März 2024

[5] Johnell O, Kanis J (2005) Epidemiology of osteoporotic fractures. Osteoporos Int. 10.1007/s00198-004-1702-6 15365697 10.1007/s00198-004-1702-6

[6] Court-Brown CM, McQueen MM (2016) Global Forum: Fractures in the Elderly. J Bone Joint Surg Am. 10.2106/JBJS.15.00793 27147693 10.2106/JBJS.15.00793

[7] Balasubramanian A, Zhang J, Chen L et al (2019) Risk of subsequent fracture after prior fracture among older women. Osteoporos Int. 10.1007/s00198-018-4732-1 30456571 10.1007/s00198-018-4732-1 PMC6332293

[8] Söreskog E, Ström O, Spångéus A et al (2020) Risk of major osteoporotic fracture after first, second and third fracture in Swedish women aged 50 years and older. Bone. 10.1016/j.bone.2020.115286 32070789 10.1016/j.bone.2020.115286

[9] Chen CW, Huang TL, Su LT et al (2013) Incidence of subsequent hip fractures is significantly increased within the first month after distal radius fracture in patients older than 60 years. J Trauma Acute Care Surg. 10.1097/ta.0b013e31824bb325 23505668 10.1097/ta.0b013e31824bb325

[10] El Maghraoui A, Rezqi A, Mounach A et al (2013) Systematic vertebral fracture assessment in asymptomatic postmenopausal women. Bone. 10.1016/j.bone.2012.09.023 23017663 10.1016/j.bone.2012.09.023

[11] Hernlund E, Svedbom A, Ivergård M et al (2013) Osteoporosis in the European Union: medical management, epidemiology and economic burden. A report prepared in collaboration with the International Osteoporosis Foundation (IOF) and the European Federation of Pharmaceutical Industry Associations (EFPIA). Arch Osteoporos. 10.1007/s11657-013-0136-1 24113837 10.1007/s11657-013-0136-1 PMC3880487

[12] Felsenberg D, Silman AJ, Lunt M et al (2002) Incidence of vertebral fracture in europe: results from the European Prospective Osteoporosis Study (EPOS). J Bone Miner Res 10.1359/jbmr.2002.17.4.716 11918229

[13] Thomasius F, Baum E, Bernecker P et al (2018) DVO Leitlinie 2017 zur Prophylaxe, Diagnostik und Therapie der Osteoporose bei postmenopausalen Frauen und Männern. Osteologie. 10.1055/s-0038-1673537

[14] FRAX https://www.sheffield.ac.uk/FRAX/tool.aspx?lang=de. Zugegriffen: 27. März 2024

[15] Camacho PM, Petak SM, Binkley N et al (2020) American Association Of Clinical Endocrinologists/American College Of Endocrinology Clinical Practice Guidelines For The Diagnosis And Treatment Of Postmenopausal Osteoporosis—2020 Update Executive Summary. Endocr Pract. 10.4158/GL-2020-0524 32427525 10.4158/GL-2020-0524

[16] Bundesamt S (2021) Bevölkerung und Demografie – Auszug aus dem Datenreport. https://www.destatis.de/DE/Service/Statistik-Campus/Datenreport/Downloads/datenreport-2021-kap-1.pdf?__blob=publicationFile. Zugegriffen: 9. Mai 2024

[17] Bundesamt S Sterbefälle und Lebenserwartung. https://www.destatis.de/DE/Themen/Gesellschaft-Umwelt/Bevoelkerung/Sterbefaelle-Lebenserwartung/_inhalt.html#454854. Zugegriffen: 9. Mai 2024

[18] Stumpf U, Schmidmaier R (2024) Secondary fracture prevention/Update osteoporosis guidelines 2023 of the Umbrella Organization Osteology. Unfallchirurgie. 10.1007/s00113-024-01415-3 38526813 10.1007/s00113-024-01415-3

[19] El Maghraoui A, Sadni S, Jbili N et al (2014) The discriminative ability of FRAX, the WHO algorithm, to identify women with prevalent asymptomatic vertebral fractures: a cross-sectional study. BMC Musculoskelet Disord. 10.1186/1471-2474-15-365 25366306 10.1186/1471-2474-15-365 PMC4226884

